# TRIM34 modulates influenza virus-activated programmed cell death by targeting Z-DNA-binding protein 1 for K63-linked polyubiquitination

**DOI:** 10.1016/j.jbc.2022.101611

**Published:** 2022-01-20

**Authors:** Xiaoyan Wang, Jing Xiong, Diwei Zhou, Shanfeng Zhang, Li Wang, Qingqing Tian, Changming Li, Jie Liu, Yaping Wu, Junying Li, Jun Wang

**Affiliations:** 1 Department of Pathology, Tongji Hospital, Tongji Medical College, Huazhong University of Science and Technology, Wuhan, China; 2 Department of Pathology, Union Hospital, Tongji Medical College, Huazhong University of Science and Technology, Wuhan, China; 3 Department of Orthopedics, Wuhan Fourth Hospital, Puai Hospital, Tongji Medical College, Huazhong University of Science and Technology, Wuhan, China; 4 Department of Stomatology, Tianyou Hospital affiliated to Wuhan University of Science and Technology, Wuhan, China; 5 Department of Pathology, The Central Hospital of Wuhan, Tongji Medical College, Huazhong University of Science and Technology, Wuhan, China; 6 Department of Oncology, Tongji Hospital, Tongji Medical College, Huazhong University of Science and Technology, Wuhan, China; 7 Department of Gastrointestinal Surgery, Tongji Hospital, Tongji Medical College, Huazhong University of Science and Technology, Wuhan, China

**Keywords:** influenza A virus, programmed cell death, ZBP1, TRIM34, polyubiquitination, BMDM, bone marrow–derived macrophages, Co-IP, co-immunoprecipitation, FBS, fetal bovine serum, GSDMD, gasdermin D, IAV, influenza A virus, IFN, interferon, IgG, immunoglobulin G, IL, interleukin, KO, knockout, MCMV, murine cytomegalovirus, PBMC, peripheral blood mononuclear cell, RHIM, receptor-interacting protein kinase homotypic interaction motif, RSV, respiratory syncytial virus, SeV, Sendai virus, TNF-α, tumor necrosis factor-α, TRIM, tripartite motif, VSV, vesicular stomatitis virus, ZBP1, Z-DNA-binding protein 1

## Abstract

Z-DNA-binding protein 1 (ZBP1) is an innate sensor of influenza A virus (IAV) that participates in IAV-induced programmed cell death. Nevertheless, little is known about the upstream signaling pathways regulating ZBP1. We found that a member of the tripartite motif (TRIM) family, TRIM34, interacted with ZBP1 to promote its K63-linked polyubiquitination. Using a series of genetic approaches, we provide *in vitro* and *in vivo* evidence indicating that IAV triggered cell death and inflammatory responses *via* dependent on TRIM34/ZBP1 interaction. TRIM34 and ZBP1 expression and interaction protected mice from death during IAV infection owing to reduced inflammatory responses and epithelial damage. Additionally, analysis of clinical samples revealed that TRIM34 associates with ZBP1 and mediates ZBP1 polyubiquitination *in vivo*. Higher levels of proinflammatory cytokines correlated with higher levels of ZBP1 in IAV-infected patients. Taken together, we conclude that TRIM34 serves as a critical regulator of IAV-induced programmed cell death by mediating the K63-linked polyubiquitination of ZBP1.

Influenza A virus (IAV) belongs to the family Orthomyxoviridae. It is a highly contagious single-stranded RNA virus that causes respiratory tract infections and acute severe pneumonia (1). During IAV infection, some viral components are recognized by several pathogen recognition receptors, including nucleotide and oligomerization domain, leucine-rich repeat-containing proteins (NLRs), retinoic acid-inducible gene I-like receptors, and toll-like receptors (2, 3). After sensing of IAV components, cell hosts trigger multiple intracellular signaling cascades that coordinately regulate interferons (IFNs) and production of proinflammatory cytokines (2). Apart from IFN induction and proinflammatory responses, IAV sensing by innate receptors also triggers cell death, which modulates viral replication (4, 5). Nevertheless, uncontrolled epithelial cell death can exacerbate tissue injury and compromise pulmonary function (6). Several lines of evidence suggested that IAV-regulated programmed cell death occurs in at least three ways, including apoptosis, necroptosis, and pyroptosis (7, 8, 9). Uncontrolled apoptosis and necroptosis in airway epithelial cells exacerbates pneumonia and mortality during IAV infection (10). Unlike apoptosis and necroptosis, NLRP3 inflammasome activation results in pyroptosis and release of interleukin-1β (IL-1β) and IL-18, which protects cells in response to acute IAV infection (11).

Z-DNA-binding protein (ZBP1), also known as DAI (DNA-dependent activator of IFN regulatory factors), was recently identified as the first cytosolic DNA receptor (12). Nevertheless, DAI is not essential for IFN responses to most DNA viruses (13). The ZBP1 protein constitutes two receptor-interacting protein kinase homotypic interaction motif (RHIM) domains at its C terminus, with two Zα domains at its N terminus (14, 15). The RHIM domain of ZBP1 protein physically interacts with other RHIM-containing proteins (14, 15). The Zα domain of ZBP1 protein selectively binds B-DNA and left-handed double-helical ‘‘Z-form’’ RNA structures (14, 15). ZBP1 interacts with nucleic acid in the cytoplasm, and this induces interferon regulatory factor and NF-κB activation, as well as the subsequent induction of IFNs and proinflammatory cytokines (16, 17). Activated ZBP1 also recruits RIPK3, leading to programmed cell death (16, 17). The critical role of ZBP1 in inducing programmed cell death is well established; nevertheless, the activation mechanisms of ZBP1 remain largely unknown.

Tripartite motif (TRIM) family includes over 70 highly conserved proteins, most of which contain a RING domain, a B-box domain, and a predicted coiled coil domain (18). Members of the TRIM family usually have E3 ubiquitin ligase activity and function in cellular processes including intracellular signaling, innate immunity, autophagy, and carcinogenesis (19). Although functions of several TRIM family members have been described, no clear function of TRIM34 has been defined. In this study, we identified a previously unknown role of TRIM34 in IAV-activated programmed cell death. We demonstrated that TRIM34 targeted ZBP1 for K63-linked polyubiquitination. As a result, TRIM34 promoted IAV-activated programmed cell death *via* activation of the ZBP1 downstream signaling pathway.、

## Results

### Identification of TRIM34 as a ZBP1-associated protein

**Figure 1:**
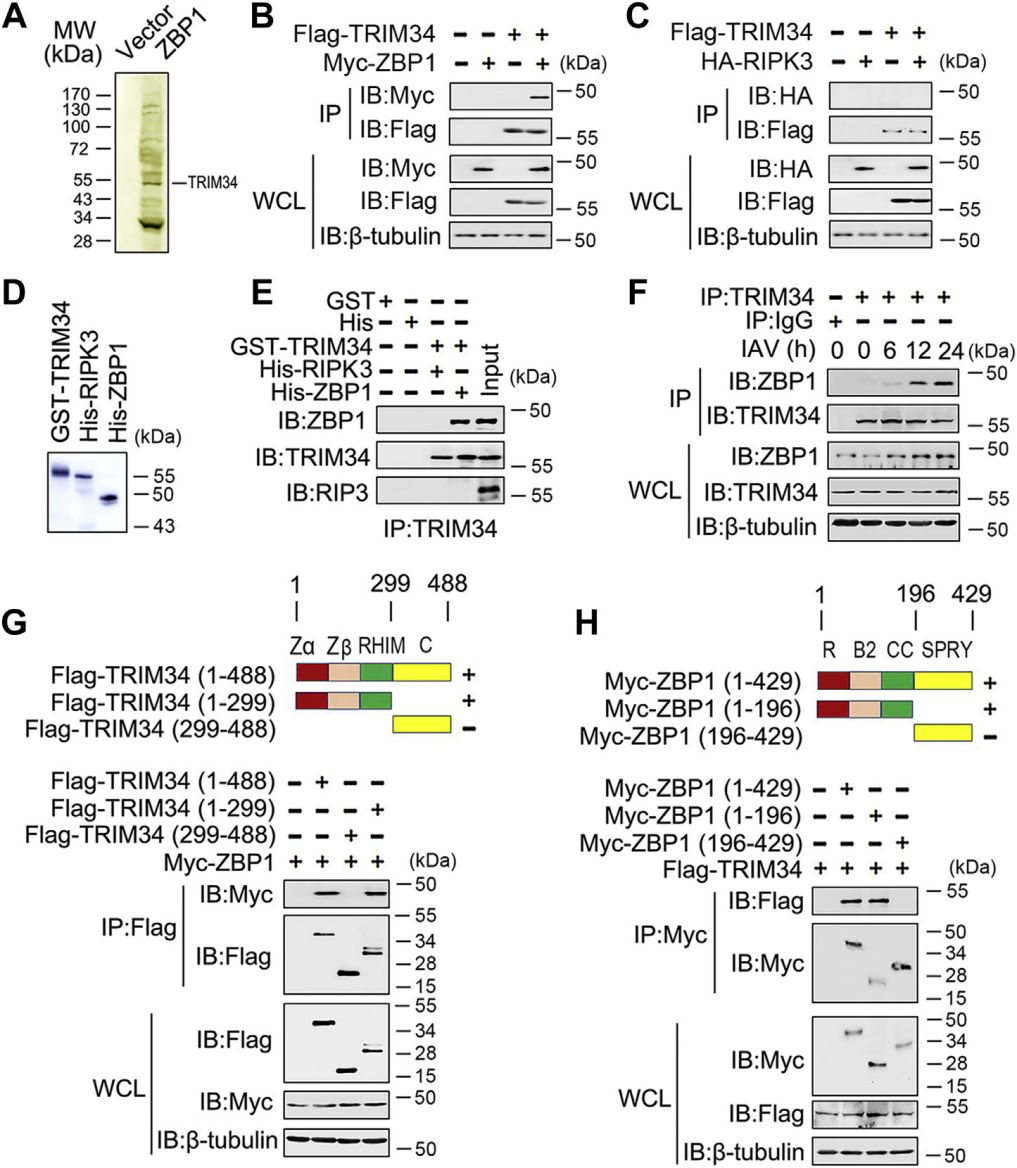
**TRIM34 interacts with ZBP1.***A*, THP-1 macrophages were transfected with vector control or Myc-ZBP1 for 48 h. Cells were lysed and were immunopurified with anti-myc affinity columns and eluted with myc peptide. The elutes were resolved using SDS-PAGE and were silver stained. Different protein bands were retrieved and analyzed using mass spectrometry. *B* and *C*, 293T cells were transfected with Myc-ZBP1 and Flag-TRIM34 (*B*) or HA-RIPK3 (*C*). Forty-eight hours post-transfection, Co-IP and immunoblot analysis were performed with the indicated antibodies. *D* and *E*, purified GST-TRIM34, His-RIPK3, and His-ZBP1 were mixed (3.2 nM) in various combinations as indicated, and the solution was immunoprecipitated with indicated antibody and analyzed using Western blotting. *F*, PBMCs were infected with IAV (multiple of infection = 1) for the indicated times or left uninfected. Co-IP and immunoblot analysis were performed with the indicated antibodies. *G*, schematic diagram of the full-length and truncated constructs of TRIM34 (*upper panel*). 293T cells were co-transfected with Myc-ZBP1 and the indicated truncated TRIM34 constructs for 48 h. Co-IP and immunoblot analyses were performed with the indicated antibodies (*lower panel*). *H*, experiments were performed similar to those in (*G*), except indicated truncated constructs of ZBP1 were used. All experiments were repeated at least three times. B1, B-box domain 1; B2, B-box domain 2; C, C-terminal domains; Co-IP, co-immunoprecipitation; IAV, influenza A virus; R, RING-finger domain; RHIM, Receptor-interacting protein kinase homotypic interaction motif domain; SPRY, SPRY domain; TRIM, tripartite motif; ZBP1, Z-DNA-binding protein 1.

To explore the molecular mechanisms of ZBP1-regulated innate immune responses, we used immunoprecipitation coupled to mass spectrometry to identify binding partners for ZBP1. We found that ZBP1 was a potential target of TRIM34 (Fig. 1*A*). Co-immunoprecipitation (Co-IP) and reverse Co-IP experiments further confirmed the binding of the TRIM34 to ZBP1. As shown in Figure 1*B*, Flag-tagged TRIM34 interacted with Myc-tagged ZBP1. Conversely, another ZBP1-associated protein, RIPK3, did not interact with TRIM34 (Fig. 1*C*). The findings were corroborated by *in vitro* binding assays using purified proteins (Fig. 1, *D* and *E*). In addition, another Co-IP experimental design also showed that ZBP1 interacts with TRIM34, but not RIPK3 (Fig. S1). Further endogenous Co-IP experiments showed that ZBP1 weakly associated with TRIM34 in untreated cells, and this association increased after stimulation with IAV (Fig. 1*F*). To map the region of TRIM34 that interacted with ZBP1, we constructed a series of TRIM34 plasmids with Flag-tagged truncation mutants (Fig. 1*G*, upper panel). We found that the N-terminal domain of TRIM34 was necessary for its interaction with ZBP1 (Fig. 1*G*, lower panel). Similarly, the N-terminal domain of ZBP1 was required for its binding to TRIM34 (Fig. 1*H*).

**Figure 2:**
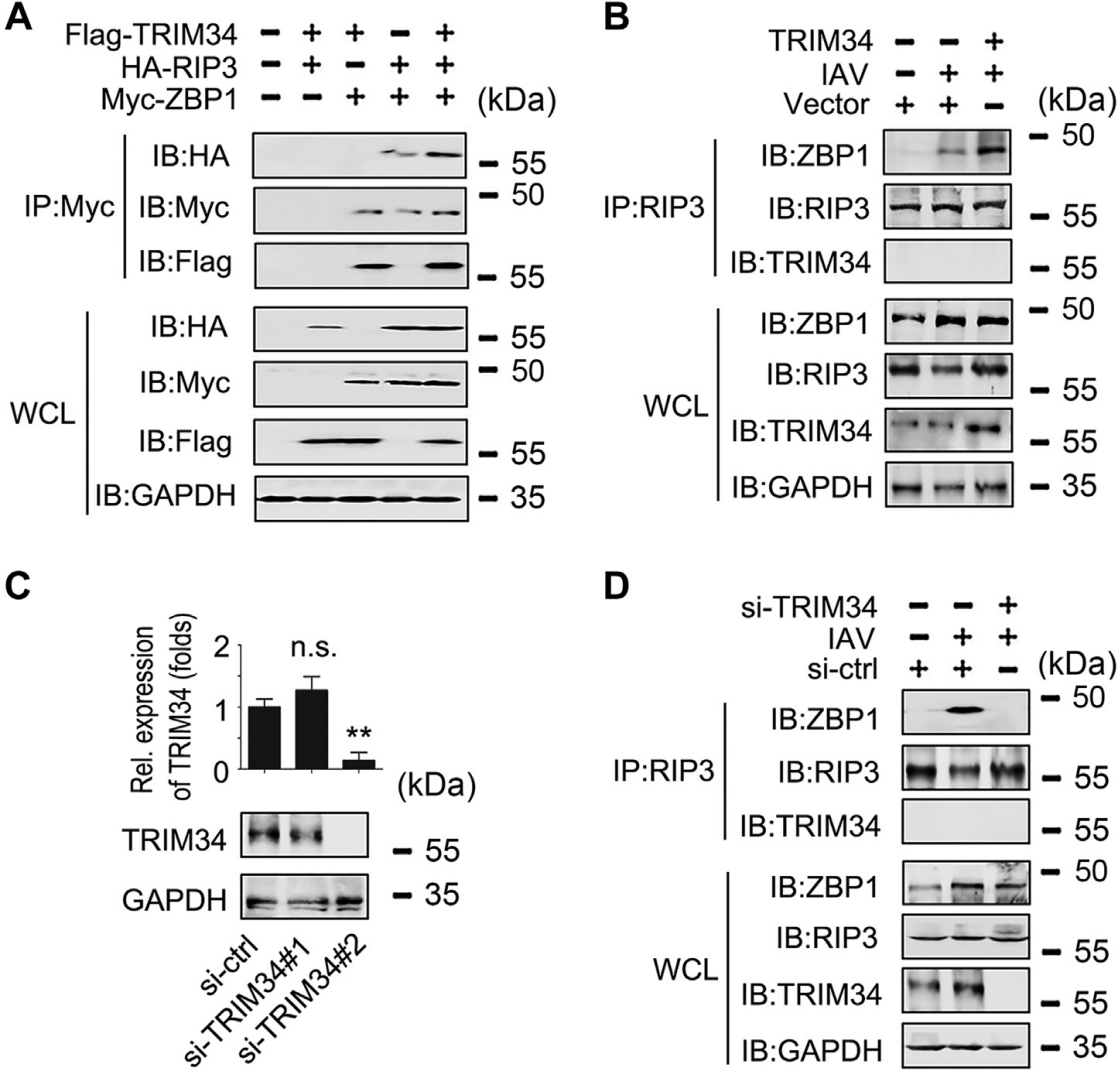
**TRIM34 promotes the interaction between ZBP1 and RIP3.***A*, 293T cells were transfected with indicated ubiquitin plasmids. Forty-eight hours post-transfection, Co-IP and immunoblot analysis were performed with indicated antibodies. *B*, THP-1 macrophages were transfected with vector control or Flag-TRIM34 for 24 h. Then, cells were infected with IAV (MOI = 1) for 24 h or left uninfected. Co-IP and immunoblot analysis were performed with the indicated antibodies. *C*, THP-1 macrophages were transfected with siRNA-control (si-ctrl) or siRNA-TRIM34 (*right panel*) for 48 h prior to qPCR (*upper panel*) and Western blot assays (*lower panel*). *D*, THP-1 macrophages were transfected with siRNA-control (si-ctrl) or siRNA-TRIM34 (*right panel*) for 24 h. Then, cells were infected with IAV (MOI = 1) for 24 h or left uninfected. Co-IP and immunoblot analysis were performed with the indicated antibodies. All experiments were repeated at least three times. Bar graphs present means ± SD (∗∗*p* < 0.01, n.s., not significant). Co-IP, co-immunoprecipitation; IAV, influenza A virus; qPCR, quantitative RT-PCR; TRIM, tripartite motif; ZBP1, Z-DNA-binding protein 1.

Because ZBP1 interacts with RIPK3 to mediate virus-induced programmed necrosis (14), we investigated the role of TRIM34 on ZBP1/RIPK3 interaction. As shown in Figure 2*A*, TRIM34 promoted ZBP1/RIPK3 interaction. Further endogenous Co-IP experiments indicated that TRIM34 overexpression enhanced IAV-induced ZBP1/RIPK3 interaction (Fig. 2*B*). We then designed two specific siRNAs for TRIM34 (siRNA-TRIM34 #1 and siRNA-TRIM34 #2) and tested their efficiency (Fig. 2*C*). As expected, TRIM34 knockdown abolished the IAV-induced ZBP1/RIPK3 interaction (Fig. 2*D*). Taken together, these data suggest that TRIM34 is associated with ZBP1, and this association promotes ZBP1 recruitment of RIPK3.

### TRIM34 induces polyubiquitination of ZBP1

**Figure 3:**
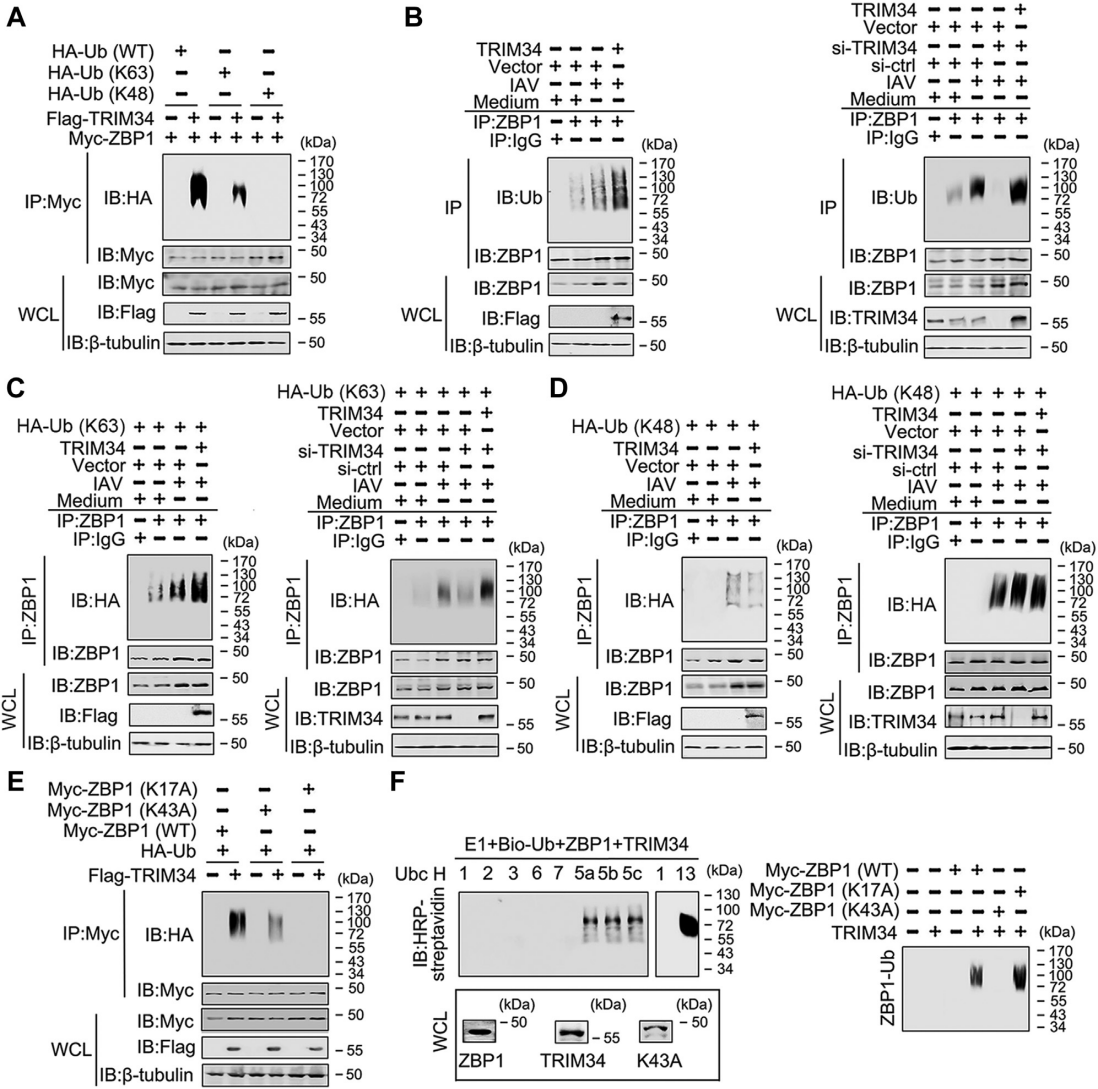
**TRIM34 promotes IAV induced K63-linked polyubiquitination of ZBP1.***A*, 293T cells were transfected with Flag-TRIM34 or/and Myc-ZBP1 and the indicated ubiquitin plasmids for 48 h. Co-IP and immunoblot analyses were performed with the indicated antibodies. *B*, THP-1 macrophages were transfected with plasmids or siRNAs for 24 h. Then, cells were infected with IAV (MOI = 1) for 24 h or left uninfected. Co-IP and immunoblot analysis were performed with the indicated antibodies. *C* and *D*, experiments were performed similar to those in (*B*), except HA-Ub (K63) plasmid (*C*) or HA-Ub (K48) plasmid (*D*) were used. *E*, 293T cells were transfected with the indicated plasmids for 48 h. Co-IP and immunoblot analyses were performed with the indicated antibodies. *F*, ZBP1, ZBP1 (K17A), ZBP1 (K43A), and TRIM34 were translated *in vitro*, and biotin-ubiquitin, E1, and the indicated E2s were added for ubiquitination assays. Ubiquitin-conjugated proteins were detected by immunoblot with horseradish peroxidase-streptavidin. Before ubiquitination analysis, the input levels of the translated proteins were detected by immunoblots. All experiments were repeated at least three times. IAV, influenza A virus; TRIM, tripartite motif; ZBP1, Z-DNA-binding protein 1.

Because many TRIM family members have E3 ubiquitin ligase activity (18), we tested whether TRIM34 regulated polyubiquitination of ZBP1. As shown in Figure 3*A*, TRIM34 enhanced K63-linked, but not K48-linked, polyubiquitination of ZBP1. Endogenous Co-IP experiments indicated that TRIM34 overexpression induced IAV-regulated ZBP1 polyubiquitination, whereas TRIM34 knockdown reduced IAV-regulated ZBP1 polyubiquitination (Fig. 3*B*). We next determined whether TRIM34 mediated K48- or K63-linked endogenous polyubiquitination of ZBP1. TRIM34 overexpression promoted IAV-induced K63-linked polyubiquitination of ZBP1, whereas TRIM34 knockdown inhibited IAV-induced K63-linked polyubiquitination of ZBP1 (Fig. 3*C*). Nevertheless, neither overexpression nor knockdown of TRIM34 affected IAV-induced K48-linked polyubiquitination of ZBP1 (Fig. 3*D*).

**Figure 4:**
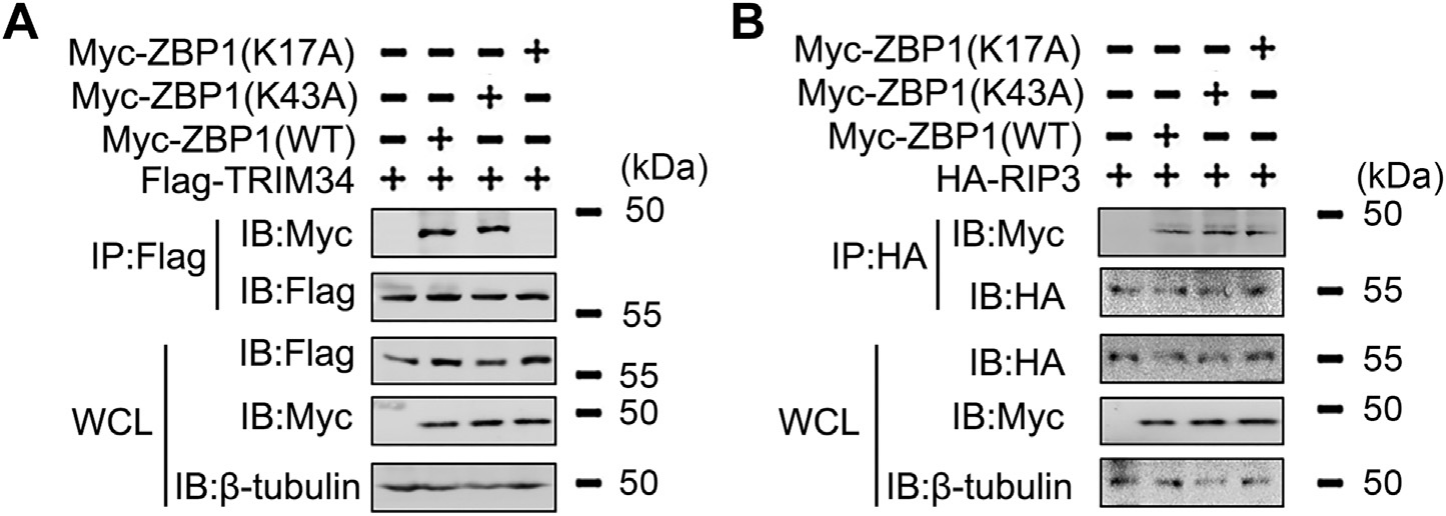
**The role of ZBP1 K17 and K43 positions on the ZBP1/TRIM34 and ZBP1/RIP3 interaction.***A* and *B*, 293T cells were transfected with indicated plasmids for 48 h. Co-IP and immunoblot analysis were performed with indicated antibodies. All experiments were repeated at least three times. Co-IP, co-immunoprecipitation; IAV, influenza A virus; TRIM, tripartite motif; ZBP1, Z-DNA-binding protein 1.

It has been verified that polyubiquitination of the K17 and K43 residue of ZBP1 is required for ZBP1 activation (16). Interestingly, TRIM34 failed to mediate polyubiquitination of ZBP1 (K17A), but not polyubiquitination of ZBP1 (K43A) (Fig. 3*E*). *In vitro* ubiquitination assays with UbcH5b and Ubc13 as an E2 suggested that TRIM34 ubiquitinates ZBP1 (K43A), but not ZBP1 (K17A) (Fig. 3*F*). Co-IP experiments indicated that TRIM34 interacts with ZBP1 (K43A), but not ZBP1 (K17A) (Fig. 4*A*). By contrast, both ZBP1 (K43A) and ZBP1 (K17A) associated with RIPK3 (Fig. 4*B*). Taken together, these data suggest that TRIM34 mediates K63-linked polyubiquitination of ZBP1 at residue K17.

### TRIM34 regulates ZBP1 the downstream signaling pathway during IAV infection *in vitro*

**Figure 5:**
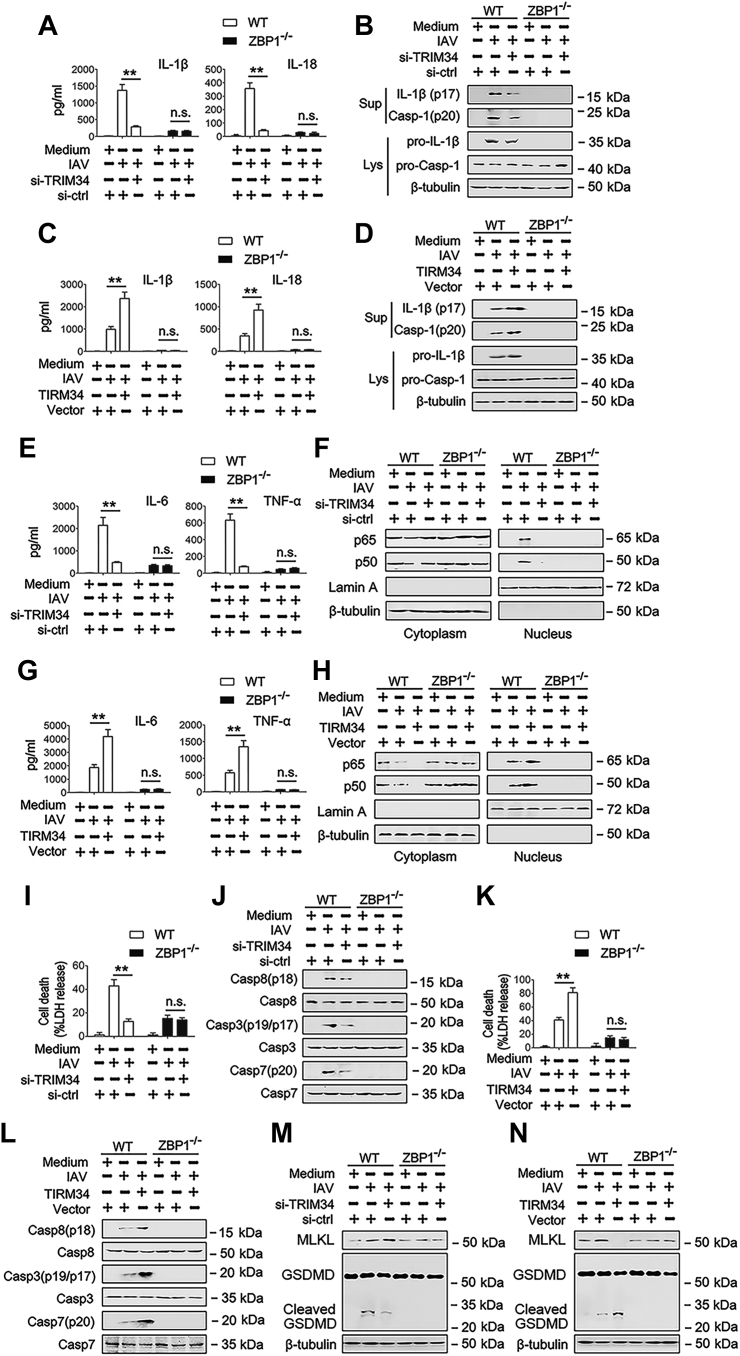
**TRIM34 regulates IAV induced NLRP3 inflammasome and programmed cell death pathways *via* ZBP1 *in vitro*.***A*, scramble-KO THP-1 macrophages (ZBP1^+/+^) and ZBP1-deficient THP-1 macrophages (ZBP1^−/−^) were transfected with siRNA-control (si-ctrl) or siRNA-TRIM34 for 24 h. Then, cells were infected with IAV (MOI = 1) for 24 h or left uninfected. IL-1β and IL-18 protein levels in cell culture supernatants were determined by ELISA. *B*, ZBP1^+/+^ and ZBP1^−/−^ THP-1 macrophages were transfected with siRNA-control (si-ctrl) or siRNA-TRIM34 for 24 h. Then, cells were infected with IAV (MOI = 1) for 24 h or left uninfected. Mature IL-1β and p20 in supernatants or pro-IL-1β and pro-Casp-1 in lysates were determined by Western blot. *C* and *D*, experiments were performed as described in (*A*) and (*B*), except that cells were transfected with vector control or Flag-TRIM34. *E*, ZBP1^+/+^ and ZBP1^−/−^ THP-1 macrophages were transfected with siRNA-control (si-ctrl) or siRNA-TRIM34 for 24 h. Then, cells were infected with IAV (MOI = 1) for 24 h or left uninfected. IL-6 and TNF-α protein levels in cell culture supernatants were determined by ELISA. *F*, experiments were performed as described in (*E*), except that cytosolic and nuclear extracts were prepared and subjected to Western blot analyses. Lamin A and β-tubulin were used as markers for nuclear and cytosolic fractions, respectively. *G* and *H*, experiments were performed as described in (*E*) and (*F*), except that cells were transfected with vector control or Flag-TRIM34. *I*, ZBP1^+/+^ and ZBP1^−/−^ THP-1 macrophages were transfected with siRNA-control (si-ctrl) or siRNA-TRIM34 (*right panel*) for 24 h. Then, cells were infected with IAV (MOI = 1) for 24 h or left uninfected. Cell death was quantified by LDH release. *J*, experiments were performed as described in (*I*), except that the pro and cleaved forms of caspase-8, caspase-3, and caspase-7 were determined by Western blot. *K* and *L*, experiments were performed as described in (*I*) and (*J*), except that the cells were transfected with vector control or Flag-TRIM34. *M* and *N*, experiments were performed as described in (*J*) and (*L*), except that MLKL and GSDMD were determined by Western blot. All experiments were repeated at least three times. Bar graphs present means ± SD, n = 3 (∗∗*p* < 0.01, n.s., not significant). GSDMD, gasdermin D; IAV, influenza A virus; KO, knockout; MLKL, mixed-lineage kinase domain-like protein; TRIM, tripartite motif; ZBP1, Z-DNA-binding protein 1.

**Figure 6:**
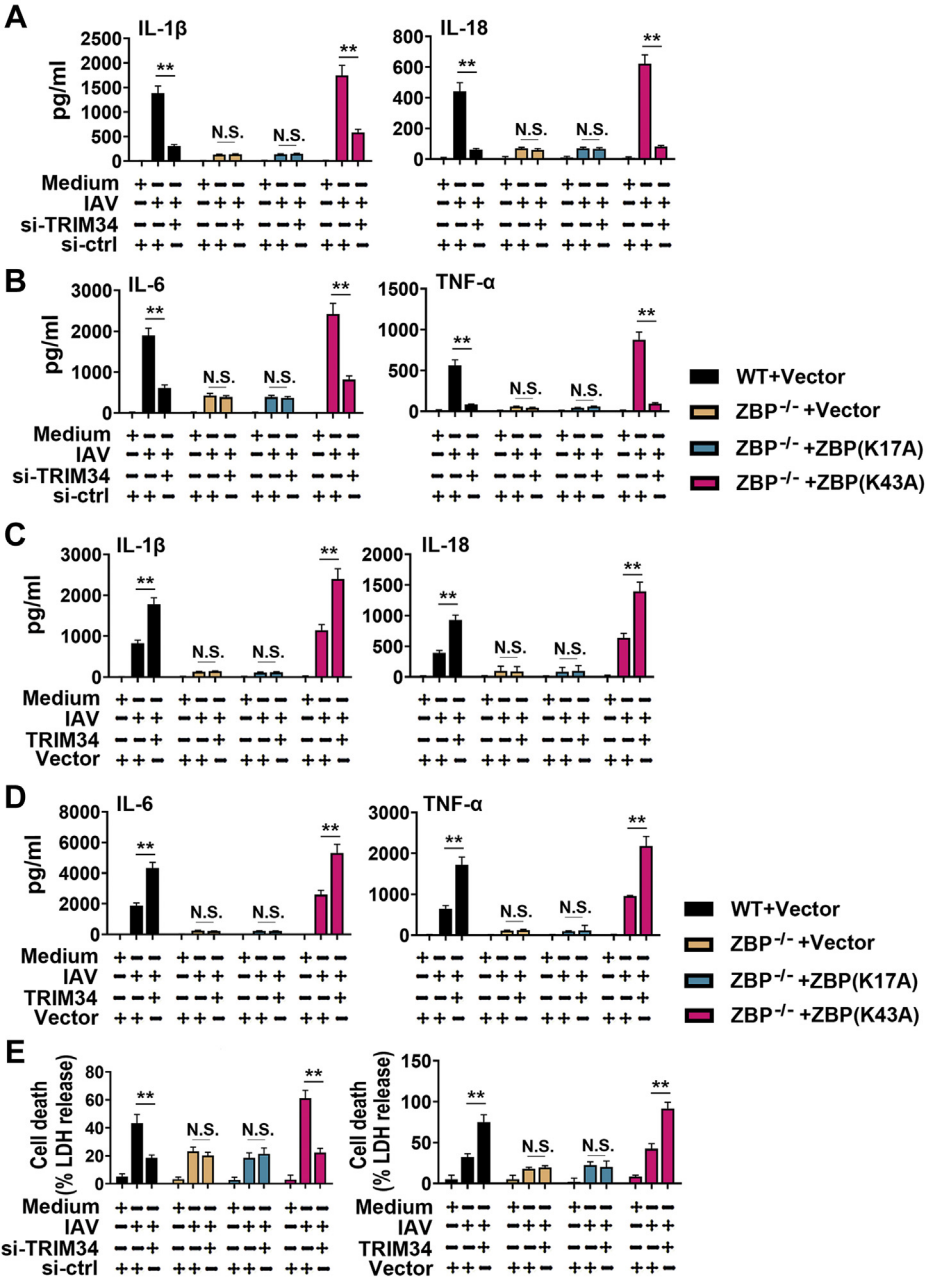
**The ZBP-1 K43A mutant, but not the ZBP-1 K17A mutant, rescues ZBP-1 function in ZBP-1 knockout cells.***A* and *B*, ZBP1^+/+^ and ZBP1^−/−^ THP-1 macrophages were transfected with siRNA-control (si-ctrl) or siRNA-TRIM34 and indicated plasmids for 24 h. Then, cells were infected with IAV (MOI = 1) for 24 h or left uninfected. IL-1β, IL-18, IL-6, and TNF-α protein levels in cell culture supernatants were determined by ELISA. *C* and *D*, experiments were performed as described in (*A*) and (*B*), except that the cells were transfected with vector control or Flag-TRIM34. *E*, experiments were performed as described in (*A*) and (*C*), except that cell death was quantified by LDH release. All experiments were repeated at least three times. Bar graphs present means ± SD, n = 3 (∗∗*p* < 0.01; n.s., not significant). IAV, influenza A virus; TRIM, tripartite motif; ZBP1, Z-DNA-binding protein 1.

Because ZBP1 is important for IAV-induced programmed cell death pathways, including NLRP3 inflammasome activation, necroptosis, and apoptosis (17), we reasoned that TRIM34 regulates ZBP1 mediated downstream signaling pathway. First, we investigate whether TRIM34 regulated ZBP1-mediated NLRP3 inflammasome activation in response to IAV infection. As shown in Figure 5, *A* and *B* and Fig. S2*A*, TRIM34 knockdown inhibited IAV-induced caspase-1 cleavage and IL-1β and IL-18 secretion in WT cells. Nevertheless, TRIM34 knockdown did not affect IAV-induced caspase-1 cleavage and IL-1β and IL-18 secretion in ZBP1 knockout (KO) (ZBP1^−/−^) cells (Figs. 5, *A* and *B* and S2*A*). Conversely, TRIM34 overexpression enhanced IAV-induced caspase-1 cleavage and IL-1β and IL-18 secretion in WT cells, but not in ZBP1^−/−^ cells (Figs. 5, *C* and *D* and S2*B*). The ZBP1 also transduced NF-κB activation signals (17). Consistent with this, TRIM34 overexpression increased IAV-induced IL-6 and tumor necrosis factor-α (TNF-α) secretion and NF-κB translocation from the cytoplasm to the nucleus, whereas TRIM34 knockdown decreased IAV-induced IL-6 and TNF-α secretion and NF-κB translocation from the cytoplasm in WT cell, but not in ZBP1^−/−^ cells (Figs. 5, *E*–*H* and S2, *C* and *D*). Similar results were obtained in NF-κB-luc activity assays (Fig. S3*A*). Previous studies found that ZBP1 promoted apoptosis during IAV infection (17). Interestingly, knockdown of TRIM34 decreased IAV-induced cell death and activation of caspase-8, caspase-3, and caspase-7; conversely, overexpression of TRIM34 enhanced IAV-induced cell death and activation of caspase-8, caspase-3, and caspase-7 (Figs. 5, *I*–*L* and S2, *E* and *F*). Similar results were observed by testing mixed-lineage kinase domain-like protein, gasdermin D (GSDMD) and cleaved GSDMD expression (Fig. 5, *M* and *N*). Interesting, re-expression of ZBP1 (K43A), but not ZBP1 (K17A), in ZBP-1 KO cells reconstitute inflammatory factors expression during IAV infection (Fig. 6).

Given that ZBP1 was dispensable for the canonical NLRP3 activator LPS plus ATP and the noncanonical NLRP3 activators in *Escherichia coli* and *Citrobacter rodentium* infection (17), we also investigated the role of TRIM34 in these canonical and noncanonical NLRP3 activators. As shown in Fig. S3, *B*–*K*, neither overexpression nor knockdown of TRIM34 affected LPS, LPS plus ATP, HSV-1, and *E. coli*- and *Citrobacter*-induced IL-1β and IL-18 secretion in WT cells and ZBP1^−/−^ cells. ZBP1 was also dispensable for poly(I:C), poly(dA:dT), and ssRNA-40-induced cell death. In accordance with these observations, neither overexpression nor knockdown of TRIM34 affected poly(I:C), poly(dA:dT), ABT-263, and ssRNA-40-induced cell death in WT cells and ZBP1^−/−^ cells (Fig. S3, *L*–*O*). Taken together, these data suggest that TRIM34 regulates NLRP3 inflammasome activation and cell death pathways during IAV infection through ZBP1.

### TRIM34 regulates ZBP1 downstream signaling pathway during IAV infection *ex vivo*

**Figure 7:**
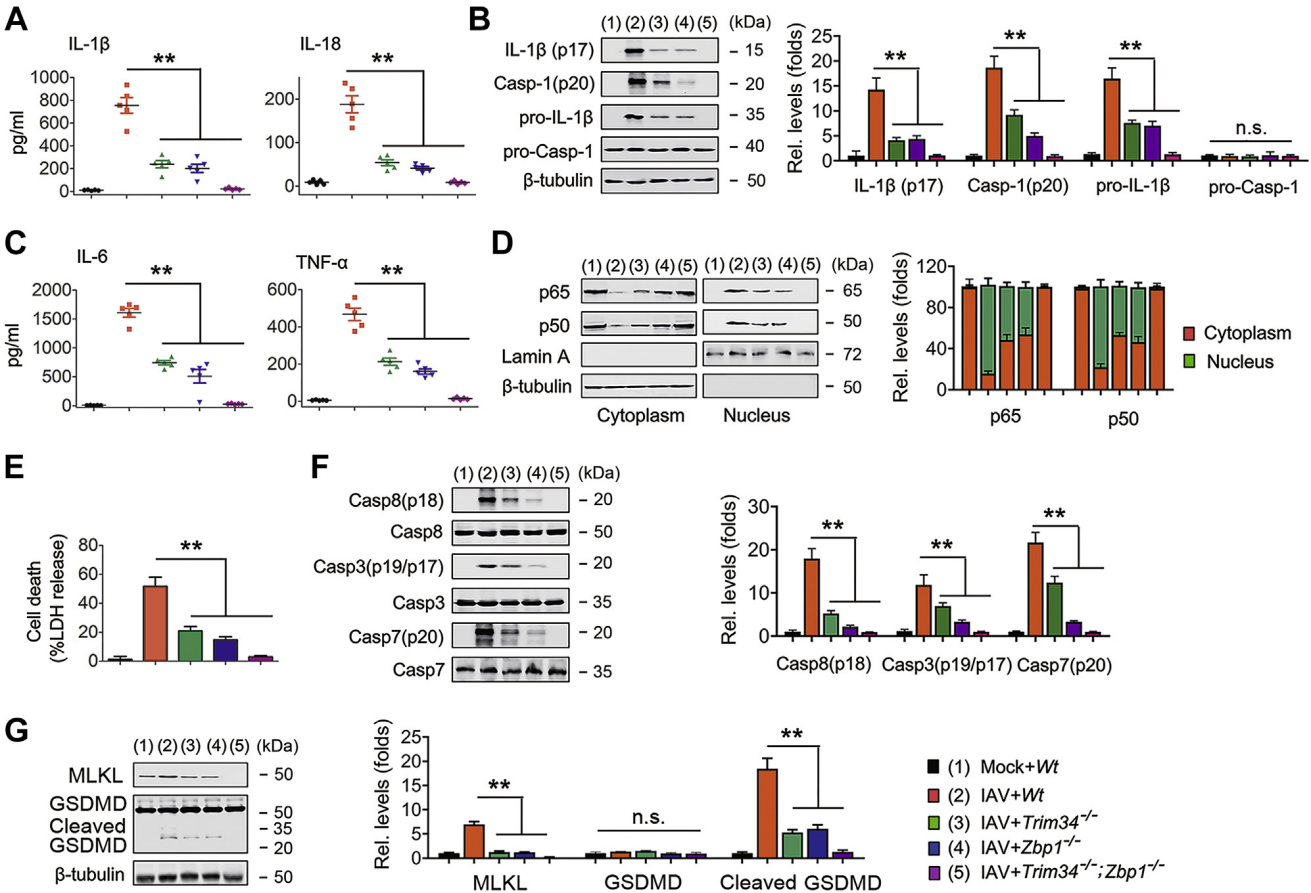
**IAV induced NLRP3 inflammasome and programmed cell death pathways via TRIM34/ZBP1 *ex vivo*.***A*, BMDMs were isolated from indicated mice and infected with IAV (MOI = 1) for 24 h or left uninfected. IL-1β and IL-18 protein levels in cell culture supernatants were determined by ELISA. *B*, BMDMs were isolated from indicated mice and infected with IAV (MOI = 1) for 24 h or left uninfected. Mature IL-1β and p20 in supernatants or pro-IL-1β and pro-Casp-1 in lysates were determined using Western blot. The IL-1β (p17)/β-tubulin, Casp-1 (p20)/β-tubulin, pro-IL-1β/β-tubulin, and pro-Casp-1/β-tubulin ratios were quantified as described above. *C*, experiments were performed as described in (*A*), except that IL-6 and TNF-α protein levels in cell culture supernatants were determined by ELISA. *D*, BMDMs from indicated mice were infected with IAV (MOI = 1) for 24 h or left uninfected. Cytosolic and nuclear extracts were prepared and subjected to Western blot analyses. Lamin A and β-tubulin were used as markers for nuclear and cytosolic fractions, respectively. Quantification of p65, p50, cytoplasmic and nuclear localization as described above. *E* and *F*, experiments were performed as described in (*A*) and (*B*), except that cell death was quantified using LDH release (*E*), or the pro and cleaved forms of caspase-8, caspase-3, and caspase-7 were determined by Western blot (*F*). The Casp8 (p18)/Casp8, Casp3 (p19/p17)/Casp3, and Casp7 (p20)/Casp7 ratios were quantified as described above. *G*, experiments were performed as described in (*E*), except that MLKL and GSDMD were determined by Western blot. The MLKL/β-tubulin, GSDMD/β-tubulin, and cleaved GSDMD/β-tubulin ratios were quantified as described above. All experiments were repeated at least three times. Bar graphs present means ± SEM, n = 3 (∗∗*p* < 0.01, n.s., not significant). GSDMD, gasdermin D; IAV, influenza A virus; MLKL, mixed-lineage kinase domain-like protein; TRIM, tripartite motif.

To further confirm the involvement of the TRIM34/ZBP1 axis in IAV-induced inflammasome activation and cell death pathways, we generated mice deficient in the *Trim34* gene (*Trim34*^−/−^ mice) using standard CRISPR/Cas9 technology (Fig. S4*A*) and purchased mice deficient in the *Zbp1* gene (*Zbp1*^−/−^ mice). *Trim34* and *Zbp1* deficiency was confirmed using immunoblot analyses (Fig. S4, *B* and *C*). We also crossed *Zbp1*^−/−^ mice with *Trim34*^−/−^ mice to establish in absence of both *Trim34* and *Zbp1* leading to the mouse line *Trim34*^−/−^;*Zbp1*^−/−^. Robust caspase-1 cleavage, IL-1β, and IL-18 secretions were observed in WT bone marrow–derived macrophages (BMDMs) infected with IAV. This response was inhibited in *Zbp1*^−/−^ BMDMs and *Trim34*^−/−^ BMDMs and almost was abrogated in *Trim34*^−/−^;*Zbp1*^−/−^ mice (Fig. 7, *A* and *B*). Similarly, IAV-induced IL-6 and TNF-α secretion and NF-κB translocation from the cytoplasm were inhibited in *Zbp1*^−/−^ BMDMs and in *Trim34*^−/−^ BMDMs (Fig. 7, *C* and *D*). IL-6 and TNF-α secretion and NF-κB translocation from the cytoplasm were completely abrogated in *Trim34*^−/−^ and *Zbp1*^−/−^ BMDMs (Fig. 7, *C* and *D*). We next tested whether IAV-regulated apoptosis was affected in BMDMs from *Trim34*^−/−^, *Zbp1*^−/−^, and *Trim34*^−/−^;*Zbp1*^−/−^ mice. In agreement with the *in vitro* data, *Trim34*^−/−^ or *Zbp1*^−/−^ BMDMs exhibited lower cell death and activation of caspase-8, caspase-3, and caspase-7, and *Trim34*^−/−^;*Zbp1*^−/−^ BMDMs exhibited the lowest cell death and activation of caspase-8, caspase-3, and caspase-7 than did WT BMDMs during IAV infection (Fig. 7, *E* and *F*). Although robust activated mixed-lineage kinase domain-like protein and cleaved GSDMD were observed in WT BMDMs infected with IAV, this response was abrogated in *Trim34*^−/−^, *Zbp1*^−/−^, and *Trim34*^−/−^;*Zbp1*^−/−^ BMDMs (Fig. 7*G*). We investigated the role of the TRIM34/ZBP1 axis in poly(I:C), poly(dA:dT), and ssRNA-40-induced cell death *ex vivo*. As shown in Fig. S3, *D*–*F*, poly(I:C), poly(dA:dT), and ssRNA-40-induced cell death was not changed in *Trim34*^−/−^, *Zbp1*^−/−^, and *Trim34*^−/−^;*Zbp1*^−/−^ BMDMs from that of WT BMDMs. Next, we investigated the role of TRIM34/ZBP1 axis on cell death *ex vivo* in response to other negative-sense RNA viruses: vesicular stomatitis virus (VSV), Sendai virus (SeV), and respiratory syncytial virus (RSV). As expected, VSV, SeV, and RSV induced comparable levels of cell death in WT, *Trim34*^−/−^, *Zbp1*^−/−^, and *Trim34*^−/−^;*Zbp1*^−/−^ BMDMs (Fig. S4, *G*–*I*). Taken together, these data suggest that TRIM34/ZBP1 axis regulates NLRP3 inflammasome activation and cell death pathways specifically in IAV infection.

### TRIM34 and ZBP1 promotes inflammatory responses and epithelial damage during IAV infection *in vivo*

**Figure 8:**
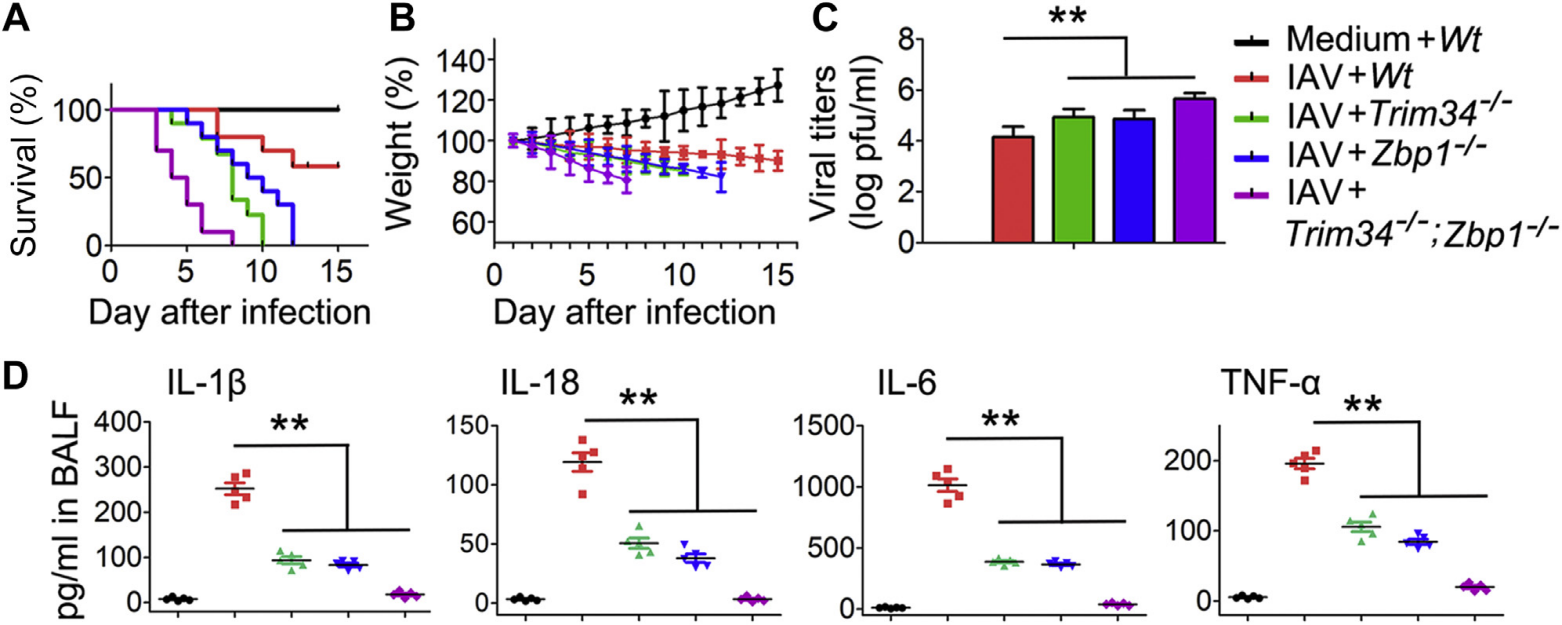
**IAV induced inflammatory responses and epithelial damage *via* TRIM34/ZBP1 *in vivo*.***A*, indicated mice (n = 10 for each group) were intranasally infected with 1 × 10^4^ pfu of IAV. Survival curves show data collected until day 14 postinfection. The statistical analysis was performed using a log-rank test. *B*, indicated mice (n = 10 for each group) were intranasally infected with 1 × 10^4^ pfu of IAV, and body weights were recorded daily. *C*, comparison of lung viral titers on day 5 post-IAV infection (n = 10 for each group). *D*, levels of proinflammatory cytokines and chemokines in bronchoalveolar lavage fluid (BALF) were measured 48 h postinfection using ELISA (n = 5 for each group). Bar graphs present means ± SEM, (∗∗*p* < 0.01, n.s., not significant). IAV, influenza A virus; TRIM, tripartite motif; ZBP1, Z-DNA-binding protein 1.

The physiological relevance of TRIM34/ZBP1 axis in regulating pathogenesis of IAV infection was assessed in WT, *Trim34*^−/−^, *Zbp1*^−/−^, and *Trim34*^−/−^;*Zbp1*^−/−^ mice. Compared with *Trim34*^−/−^ and *Zbp1*^−/−^ mice, *Trim34*^−/−^;*Zbp1*^−/−^ mice displayed significantly increased rates of mortality (Fig. 8*A*). Moreover, *Trim34*^−/−^;*Zbp1*^−/−^ mice showed the lowest body weight response to IAV infection (Fig. 8*B*). Consistent with this result, *Trim34*^−/−^ and *Zbp1*^−/−^ mice exhibited higher IAV titers, and *Trim34*^−/−^;*Zbp1*^−/−^ mice exhibited highest IAV titers than did WT mice during IAV infection (Fig. 8*C*). As expected, levels of inflammatory cytokine expression were significantly lower in bronchoalveolar lavage fluid of *Trim34*^−/−^ and *Zbp1*^−/−^ mice than in WT mice (Fig. 8*D*). Taken together, these data suggest that TRIM34/ZBP1 axis participates in regulating pathogenesis and immunopathology during acute IAV infection by controlling both cell death and inflammatory responses.

### ZBP1 correlates with inflammatory cytokine expression; TRIM34 interacts with ZBP1 for ubiquitination in patients infected with IAV

**Figure 9:**
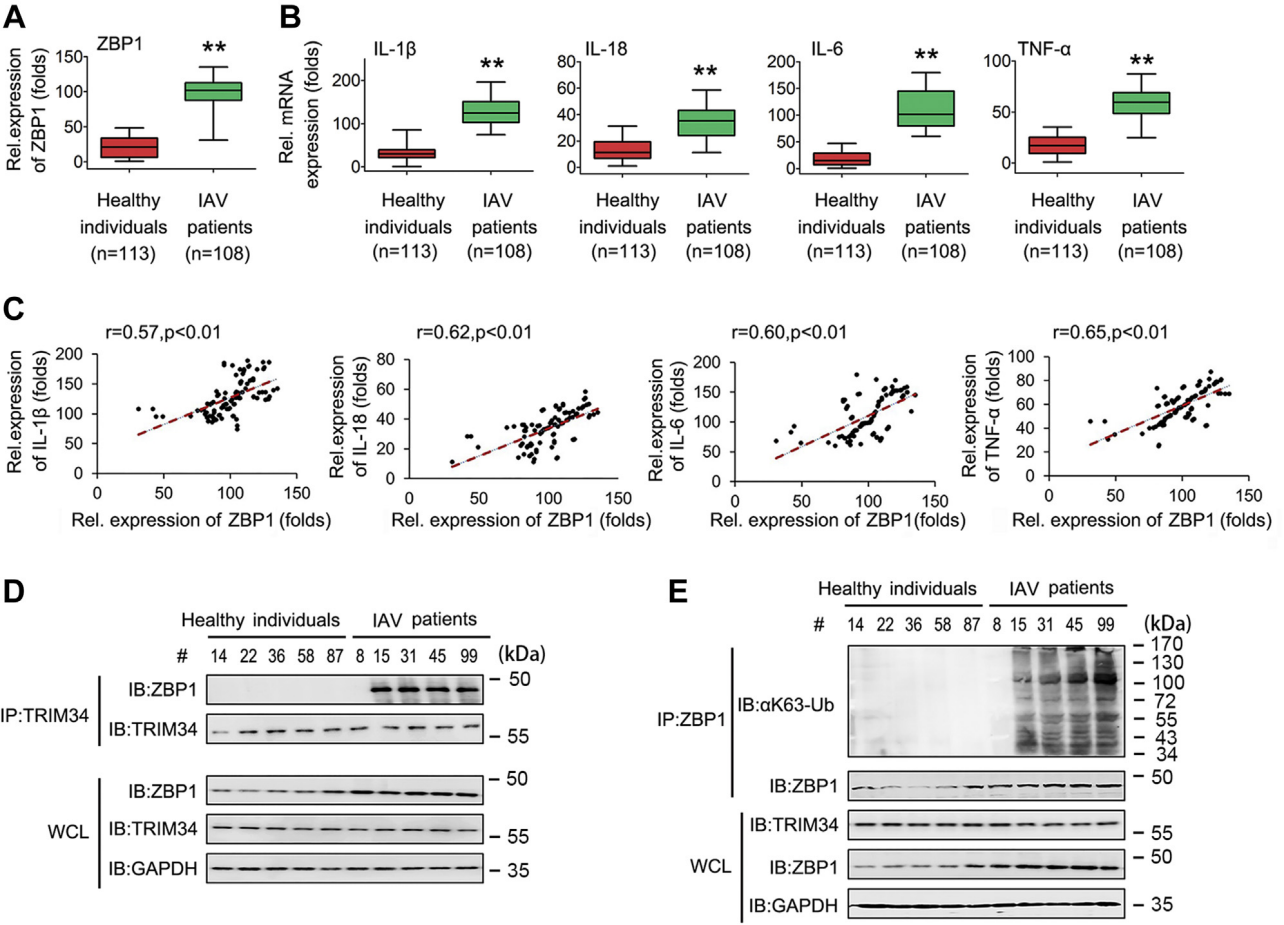
**Analysis of ZBP1, inflammatory cytokine expression, and ZBP1 ubiquitination levels in IAV-infected patients.***A* and *B*, qPCR assays of ZBP1 (*A*) and inflammatory cytokine (*B*) expression levels in PBMCs from healthy individuals (n = 113) or IAV-infected patients (n = 108). Box plots illustrate medians with 25 and 75% values, and error bars represent the 5th and 95th percentiles. Data represent means ± SEM. *C*, the relative ZBP1 mRNA and inflammatory cytokine levels in PBMCs from IAV-infected patients were subjected to Pearson’s correlation analysis. *D* and *E*, healthy individuals (#14, #22, #36, #58, #87) and IAV patients (#8, #15, #31, #45, #99) were randomly selected to isolate PBMCs. Co-IP and immunoblot analyses were performed with the indicated antibodies. (∗∗*p* < 0.01). Co-IP, co-immunoprecipitation; IAV, influenza A virus; qPCR, quantitative RT-PCR; ZBP1, Z-DNA-binding protein 1.

To further elucidate the roles of TRIM34/ZBP1 axis in IAV infection, we obtained fresh blood from healthy individuals and from patients diagnosed with IAV infection between 2017 and 2020. First, we analyze ZBP1 and inflammatory cytokine mRNA expression in healthy individuals and IAV patients. As shown in Figure 9*A*, ZBP1 mRNA levels were higher in IAV-infected patients than in healthy individuals. Accordingly, IL-1β, IL-18, IL-6, and TNF-α were expressed at significantly higher levels in IAV-infected patients than in the healthy individuals (Fig. 9*B*). Interestingly, elevated ZBP1 expression in IAV-infected patients correlated with high levels of IL-1β, IL-18, IL-6, and TNF-α (Fig. 9*C*). Next, we analyzed the interaction between ZBP1 and TRIM34 in five randomly selected healthy individuals (#14, 22, 36, 58, and 87) and five randomly selected IAV-infected patients (# 8, 15, 31, 45, and 99). Co-IP results suggested that ZBP is ubiquitylated and interacts with TRIM34 in the peripheral blood mononuclear cells (PBMCs) of IAV patients (#15, 31, 45, and 99), but not in the PBMCs of IAV patients #8 (Fig. 9, *D* and *E*). Taken together, these data suggest that ZBP1 expression levels and ZBP/TRIM34 interactions are involved in inflammatory cytokine expression in IAV-infected patients.

## Discussion

For the first time, we determined that TRIM34, a member of the TRIM family, was a positive regulator of IAV-activated programmed cell death. We found that TRIM34 targeted ZBP1 for K63-linked polyubiquitination at K17. These findings suggest a regulatory mechanism of pathogenesis and prognosis of acute influenza virus infection and might have implications for the treatment of IAV infection.

Cell fate decisions are well integrated into antiviral immune responses during IAV infection (20, 21). Several lines of evidence suggested that type I IFN signaling is vital in IAV-activated programmed cell death (22, 23). Type I IFN enhances IAV-induced apoptosis through activation of the FADD/caspase-8 death signaling pathway (24). If FADD/caspase-8 signaling was inactivated, IFNAR1/RIPK3 signaling could drive necroptosis in both mouse embryonic fibroblasts and macrophages during IAV infection (25, 26). IFN-stimulated gene factor 3 (IRF3) complex was also found to be necessary for IAV-induced necroptosis (26). A recent study found that ZBP1 is an IFN-inducible protein and located in the center of the IAV-induced apoptosis, necroptosis, and pyroptosis pathways (15, 16, 17). They demonstrated the importance of ZBP1 in driving cell death during IAV infection; nevertheless, they did not identify the upstream receptors and signaling pathways regulating ZBP1. Another interesting study found that ZBP1 ubiquitination is important in activation of programmed cell death (16). Nevertheless, the mechanisms behind this event were not identified. Here, we demonstrated that TRIM34 directly targeted ZBP1 for K63-linked polyubiquitination. It has been previously shown that K17 and K43 positions of the ZBP1 were ubiquitinated after IAV infection (16). However, in this study, we showed that TRIM34 mediated K63-linked polyubiquitination of ZBP1 at residue K17, but not at residue K43. Whether other E3 Ub ligases participate in ZBP1 ubiquitination remains unknown.

Recent advances have provided a clearer picture of immune receptors in response to IAV infection. Although the role of retinoic acid-inducible gene I in IAV infection has been studied (27), ZBP1 was only recently identified as an innate sensor of IAV. ZBP1 recognizes IAV RNA by a mechanism requiring the second of its Za domains (15). On another hand, ZBP1 also regulates murine cytomegalovirus (MCMV)-induced necroptosis (28). However, unlike IAV, MCMV is a double-stranded DNA virus. ZBP1 might sense unique patterns in ribonucleoprotein complexes formed during the transcription of MCMV genes to activate cell death. In this study, we found that both TRIM34 and ZBP1 regulate cell death in response to IAV infection. We expand the role of TRIM34/ZBP1 axis on other RNA viruses belonging to Paramyxoviridae and Rhabdoviridae families. To our surprise, the TRIM34/ZBP1 axis was dispensable for VSV-, SeV-, and RSV-induced programmed cell death. These data highlight an IAV-specific role for TRIM34/ZBP1 in initiating cell death responses. Interesting, levels of cytokines expression were lower in *Trim34*^−/−^;*Zbp1*^−/−^ BMDMs than in *Trim34*^−/−^ or *Zbp1*^−/−^ BMDMs during IAV infection (Fig. 7). This suggests that there are other factors affected by TRIM34 and ZBP1, and the phenotype is not solely explained by the TRIM34/ZBP1 interaction. When considering the next step, studies exploring these questions would be of great help in further clarifying the role of TRIM34/ZBP1 axis in IAV infection.

**Figure 10:**
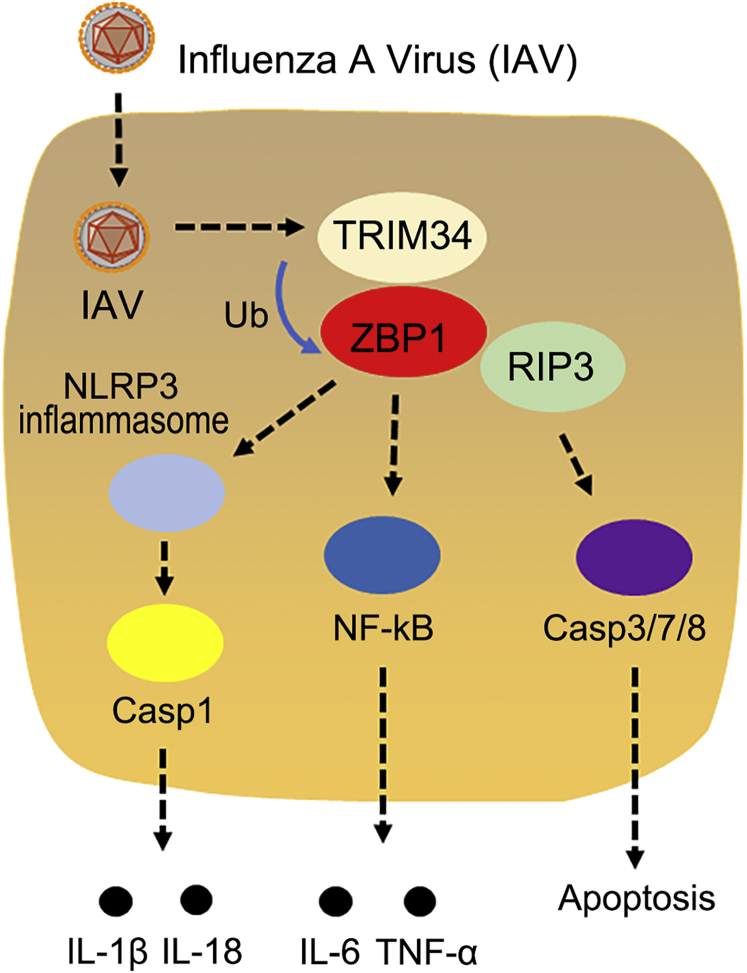
**A hypothetical model for how IAV utilizes TRIM34 to promote the NLRP3 inflammasome, NF-κB, and programmed cell death pathways by ubiquitination of ZBP1.** IAV, influenza A virus; IL, interleukin; NF-κB, nuclear factor κB; TRIM, tripartite motif; ZBP1, Z-DNA-binding protein 1.

We propose a working model describing the role of TRIM34/ZBP1 axis in IAV-activated programmed cell death (Fig. 10). In this model, TRIM34 interacts with ZBP1, leading to K63-linked ubiquitination. Subsequently, ZBP1 regulates the NLRP3 inflammasome or NF-κB activation as well as induction of the RIPK3/caspase axis. As a result, apoptosis, necroptosis, and pyroptosis were activated in IAV-infected cells. Although more studies are needed to understand the delicate regulatory mechanisms of TRIM34/ZBP1 axis in IAV-activated programmed cell death, our data provide new therapeutic strategies.

## Experimental procedures

### Clinical specimen collections

Clinical samples of patients who were positive for nasopharyngeal IAV infection were collected from Tongji Hospital of Huazhong University of Science and Technology. Blood samples from healthy individuals were randomly selected as controls from the local blood donation center. The collection of clinical samples was conducted according to the principles of the Declaration of Helsinki and was approved by the Institutional Review Board of Tongji Hospital of Huazhong University of Science and Technology in accordance with guidelines for the protection of human subjects. All study participants provided written informed consent for the collection of samples and subsequent analyses.

Fresh blood samples were drawn from healthy donors. PBMCs were obtained by density centrifugation of blood samples diluted 1:1 in pyrogen-free saline over Histopaque (Hao Yang Biotech). The separation medium, Histopaque, is a sterile-filtered, endotoxin tested solution of polysucrose and sodium diatrizoate, adjusted to a density of 1.077 g/ml. The blood-Histopaque solution was centrifuged at 800g for 20 min at room temperature, and the cell band on top of the Histopaque layer was collected. The cells were washed twice with saline and suspended in RPMI 1640 medium supplemented with penicillin (100 U/ml) and streptomycin (100 mg/ml).

### Virus and cell culture cell culture

The recombinant human IAV A/WSN/33 (H1N1) generated by transfecting MDCK cells with the eight-plasmid transfection system to generate IAV. The stock virus was propagated in 10-day-old embryonated chicken eggs (Shijun Li laboratory at Central China Agricultural University) for 36 to 48 h at 37 °C. Allantoic fluid was then harvested, and aliquots were stored at –80 °C before use. *C. rodentium* (ATCC 51459) and *E. coli* (ATCC 11775) were inoculated into LB broth and incubated overnight under aerobic conditions at 37 °C. Human embryonic kidney cells (HEK293T) were purchased from the American Type Culture Collection (ATCC, #CRL-3216). HEK293T cells were cultured in Dulbecco's modified Eagle's medium (Sigma) supplemented with 10% heat-inactivated fetal bovine serum (FBS). The human monocytic cell line THP-1 was a gift from Pro. Ying Zhu of Wuhan University. THP-1 cells were differentiated to macrophages with 60 nM tissue plasminogen activator for 12 to 14 h, and cells were cultured for 24 h without tissue plasminogen activator. THP-1 cells were cultured in RPMI-1640 medium (Sigma) supplemented with 10% heat-inactivated FBS (Sigma). Male mice BMDMs were differentiated from fresh bone marrow cells of mice. The bone marrow cells were incubated in 6-well plates for 10 days with 10% L929-conditioned, 10% heat-inactivated FBS in RPMI-1640 medium. The culture medium was replaced every 2 days. All cells were cultured at 37 °C in 5% CO2.

### Reagents and constructs

ATP, poly(I:C), poly(dA:dT), ssRNA, and LPS were purchased from Sigma-Aldrich. TRIzol, Lipofectamine-3000, normal rabbit immunoglobulin G (IgG), and normal mouse IgG, and Enzyme MIX were purchased from Invitrogen Corporation. Unless specified otherwise, all biochemical reagents were purchased from Sigma-Aldrich. Levels of lactate dehydrogenase released by cells were determined using the CytoTox 96 NonRadioactive Cytotoxicity Assay according to the manufacturer’s instructions (Promega). SiRNA-TRIM34 (stB0019689A-1-5 and stB0019689B-1-5) and negative control (siRNA-control) (siN0000001-1-5) were purchased from Ruibo Corporation. Efficiency of RNA interferences was determined using Western blotting analyses. The details of antibodies are listed in Table S1.

Mammalian expression plasmids for HA-tagged RIPK3, Myc-tagged ZBP1, Flag-tagged TRIM34 and its mutants were constructed by standard molecular biology techniques. NF-κB luciferase reporter plasmid and plasmids encoding HA-Ubi, HA-Ubi-K63, and HA-Ubi-K48 were provided by Professor Ying Zhu of College of Life Sciences at Wuhan University, China. To verify constructs and the specificity of antibodies, all constructs were transfected into 293T cells, and expression was analyzed using Western blot. All constructs were confirmed by DNA sequencing (Sangon Biotech).

### Animal studies

WT C57BL/6 mice were purchased from Shanghai Laboratory Animal Center. The mice were housed under specific pathogen-free conditions in individually ventilated cages. Mice were humanely euthanized when they met certain clinical criteria or at the end of the experiments.

For *Trim34*^−/−^ mice, we designed a pair of synthesized oligonucleotides for gRNA targeting exon 2 of TRIM34, which were annealed and cloned into the pUC57-gRNA expression vector (plasmid 51,132, Addgene) to generate the *Trim34*-deficient mouse (*Trim34*^−/−^). The gRNA expression plasmids were linearized with DraI and used as templates for *in vitro* transcription using the MEGAshortscript kit (Ambion, AM1354). The Cas9 expression plasmid (plasmid 44,758, Addgene) was linearized with AgeI and used as the template for *in vitro* transcription using the T7 Ultra Kit (Ambion, AM1345). The transcribed Cas9 mRNA and gRNA were both purified using the MEGAclear kit (Ambion, AM1908), and then a mixture of transcribed Cas9 mRNA and gRNA was microinjected into C57/BL6 mouse zygotes to generate the *Trim34*^−/−^ mouse. Microinjections were performed in the cytoplasm of zygotes using a Nikon microinjection system under standard conditions. The mouse was genotyped by PCR with the primers 5-ATTCTGTCTAGTAATGGGTGAAA-3 and 5-GGGCAAGTGGTCTTCTCCTGTAT-3. For genotyping, a 247-bp fragment of the WT allele was amplified with 30 PCR cycles consisting of 94 °C for 30 s, 60 °C for 30 s, and 72 °C for 30 s (Fig. S4*A*). The mutant *Trim34* allele 2 was used in future study.

All animal experiments were performed in accordance with the National Institutes of Health Guide for the Care and Use of Laboratory Animals. The protocol was approved by the Institutional Animal Care and Use Committee of Tongji Hospital of Huazhong University of Science and Technology. All animals were housed under specific pathogen-free conditions at 21 °C and 31% humidity.

### Generation of KO cell lines

The lentiCRISPRv2 plasmid was kindly provided by Jianguo Wu (Wuhan University). A specific oligo targeting the gene was designed using Cas9 target design tools (http://www.genome-engineering.org). The target guide sequence cloning protocol can be found at the Zhang Laboratory GeCKO Web site (http://www.genome-engineering.org/gecko/). The specific lentiCRISPRv2 plasmid, lentivirus packaging plasmid psPAX2, and envelope plasmid pMD2.G were cotransfected into 293T cells in 60-mm culture dishes using Lipofectamine 3000. The harvested medium was centrifuged at 15,000*g* for 5 min and then filtered through a 0.22-mm filter (Millipore) to remove cells. When recipient cells were grown to ∼70% confluence, they were incubated in fresh culture medium containing 8 mg/ml polybrene. Subsequently, we added specific lentiCRISPRv2 lentivirus-containing media to the cells. The monoclonal cell colonies were singled out for enlarged culture. KO cell lines were obtained from these enlarged monoclonal cells, and KO was confirmed by qRT-PCR and Western blotting.

### Quantitative RT-PCR

Total RNA was extracted using the TRIzol reagent (Invitrogen) according to the manufacturer’s instructions. Real-time quantitative RT-PCR analysis was performed using the Roche LC480 and SYBR RT-PCR kits (DBI Bioscience) in a 20-ml reaction mixture containing 0.5 mM each PCR primer, 10 ml SYBR Green PCR Master Mix, 1 ml diluted DNA template, and RNase-free water. Primers specific to either human or murine genes are listed in Tables S2. The data were normalized according to the level of β-actin expression in each sample.

### Coimmunoprecipitation

Cells were collected and lysed in IP-lysis buffer (50 mM Tris-HCl, 150 mM NaCl, 1% Triton X-100, 1 mM EDTA, 10% glycerol, and protease inhibitor cocktail, pH7.4). Supernatants were collected by centrifugation (15,000*g*, 15 min, 4 ˚C) and were precleared with 30 μl protein G-conjugated agarose (GE Healthcare Life Sciences) followed by centrifugation (2000*g*, 2 min, 4 ˚C). The precleared supernatants were incubated with the indicated antibodies (1 μg/ml) for 3 h or overnight at 4 ˚C, followed by immunoprecipitation with 30 μl protein G-conjugated agarose for 2 h at 4 ˚C. The precipitates were washed 5 to 7 times with IP-wash buffer (50 mM Tris-Cl, 300 mM NaCl, 1% Triton X-100, 1 mM EDTA, pH7.4), and bound proteins were separated by SDS-PAGE with subsequent immunoblotting analysis

### Immunopurification and mass spectrometry

Cells were transfected with the vector control or Flag-tagged TRIM34 for 48 h. Anti-Flag immunoaffinity columns were prepared using anti-FLAG M2 affinity gel (Sigma) following the manufacturer’s suggestions. Cell lysates were obtained from about 5 × 10^8^ cells and applied to an equilibrated FLAG column of 1-ml bed volume to allow for adsorption of the protein complex to the column resin. After binding, the column was washed with cold PBS plus 0.1% NP-40. Flag peptide (Sigma) was applied to the column to elute the Flag protein complex as described by the vendor. Fractions of the bottom volume were collected and resolved on SDS-polyacrylamide gel, silver stained, and subjected to LC-MS/MS sequencing and data analysis.

### Western blot analysis

Whole cell lysates were prepared by lysing cells with PBS (pH 7.4) containing 0.01% Triton X-100, 0.01% EDTA and 10% protease inhibitor mixture (Roche). The protein concentration was determined using the Bradford assay (Bio-Rad Laboratories). Cell lysates were resolved by 12% SDS–PAGE gel electrophoresis and transferred to nitrocellulose membranes (Amersham, GE Health Care). Nonspecific binding was blocked with 5% nonfat dried milk before incubation with the primary and secondary antibodies. The protein amount loaded onto the gels was 100 mg, and the protein bands were detected using SuperSignal Chemiluminescent Substrate (Pierce)

### Transfection and luciferase reporter gene assays

Cells were plated at a density of 4 × 10^5^ cells per well in 24-well or 6-well plates and grown to approximately 80% confluence at the time of transfection. The plasmids were cotransfected into the cells using the Lipofectamine 3000 reagent (Invitrogen). At 24 h post-transfection, the cells were serum starved for another 24 h prior to harvest.

The cells were lysed with the Luciferase Cell Culture Lysis reagent (Promega), and the cell lysates and luciferase assay substrate (Promega) were mixed before the light intensity was detected using a luminometer (GloMax 20/20; Promega). The assays were performed in triplicate, and the results are expressed as the mean value relative to the vector control, which was arbitrarily assigned as 100%

### Ubiquitination experiments

Cell lysates were immunoprecipitated with the indicated antibodies. The immunoprecipitates were re-extracted in lysis buffer (20 mM Tris-HCl, pH 7.4–7.5, 150 mM NaCl, 1 mM EDTA, 1% NP-40) containing 1% SDS and denatured by heating for 10 min. The samples were centrifuged at 12,000*g* for 1 min, and then the supernatants were diluted with lysis buffer until SDS concentration was decreased to 0.1%. The supernatants were then subjected to reimmunoprecipitation with the indicated antibodies. The immunoprecipitates were analyzed by immunoblots with the indicated antibodies

### Cytokine measurement

IL-1β, IL-6, TNF-α, and IL-18 were measured using enzyme-linked immunosorbent assay kits (Millipore) according to the manufacturers’ instructions.

### Histology and immunohistochemistry

Immunohistochemical staining of formalin-fixed paraffin-embedded samples was performed as described previously (1). Briefly, the formalin-fixed paraffin sections were deparaffinized, rehydrated, and pretreated with 3% H_2_O_2_ for 20 min. The antibody-binding epitopes of the antigens were retrieved using microwave treatment, and the sections were then preincubated with 10% goat serum to block nonspecific binding. The specimens were incubated with the primary antibodies for 1 h at room temperature, and IgG was used as isotype control. Sections were then incubated with anti-rabbit or anti-mouse secondary antibody and streptavidin-horseradish peroxidase. 3,3-diaminobenzidine was used as a chromogen, and hematoxylin was used for counterstaining. Positively stained areas were quantified in 6 to 10 random fields ( × 100, × 200 or × 400) on each slide using Image J software, and values are depicted in bar graphs next to the images.

### Nuclear extraction

Cells were incubated in serum-free media for 24 h, washed twice with cold PBS, and scraped into 1 ml cold PBS. Cells were harvested by centrifugation (15 s) and incubated in two packed cell volumes of buffer A (10 mM Hepes, pH 8, 0.5% Nonidet P-40, 1.5 mM MgCl_2_, 10 mM KCl, 0.5 mM DTT, and 200 mM sucrose) for 5 min at 4 ˚C with flipping of the tube. The crude nuclei were collected by centrifugation (30 s); pellets were rinsed with buffer A, resuspended in one packed cell volume of buffer B (20 mM Hepes, pH 7.9, 1.5 mM MgCl_2_, 420 mM NaCl, 0.2 mM EDTA, and 1.0 mM DTT), and incubated on a shaking platform for 30 min at 4 ˚C. Nuclei were centrifuged (5 min), and supernatants were diluted 1:1 with buffer C (20 mM Hepes, pH 7.9, 100 mM KCl, 0.2 mM EDTA, 20% glycerol, and 1 mM DTT). Cocktail protease inhibitor tablets were added to each type of buffer. Nuclear extracts were snap frozen in liquid nitrogen and stored at −70 ˚C until use.

### Protein purification

For *in vitro* pull-down assays, TRIM34 was cloned into the expression vector pGEX-4T-1 (Amersham Pharmacia) and was expressed in Rosetta (DE3) pLys (Novagen) *E. coli* cells. The recombinant proteins were further purified on Glutathione Sepharose bead (Pharmacia) columns to obtain relatively pure GST-TRIM34. His-ZBP1 and His-RIPK3 were induced in the same manner and purified on the Ni-NTA columns (Qiagen). For the *in vitro* pull-down assays, GST-TRIM34, His-ZBP1, and His-RIPK3 were incubated together in various combinations. After short incubation, the reaction systems were immunoprecipitated using agarose-immobilized indicated antibodies or protein A/G agarose beads and were then analyzed by Western blotting.

### Statistical analysis

The data presented in this article were obtained from three independent reproducible experiments. Data were presented as the mean ± standard deviations or mean ± the standard error of the mean. Student’s *t* test was performed for statistical comparisons between two groups. A one-way analysis of variance was used to compare three or more groups. Kaplan–Meier analysis was used for the survival analysis, and significance was determined using the log-rank test. A *p*-value < 0.05 was considered significant and was indicated with an asterisk symbol (∗).

## Data availability

All data are contained in the manuscript.

## Supporting information

This article contains supporting information.

## Conflict of interest

The authors have declared that no conflict of interest exists.
