# Supplementary material for: TRIM34 modulates influenza virus-activated programmed cell death by targeting Z-DNA-binding protein 1 for K63-linked polyubiquitination
Source: J Biol Chem. 2022 Jan 20;298(3):101611. doi: 10.1016/j.jbc.2022.101611 (PMC8867111; doi:10.1016/j.jbc.2022.101611)
Supplement: Supplemental Figures S1–S4 and Tables S1–S2 [file mmc1.pdf]

# Supporting Materials

# TRIM34 modulates influenza virus-activated programmed cell death through targeting ZBP1 for K63-linked polyubiquitination

Xiaoyan Wang[1], Jing Xiong[1], Diwei Zhou[2], Shanfeng Zhang[3], Li Wang[4], Qingqing Tian[5], Changming Li[1], Jie Liu[1], Yaping Wu[6], Junying Li[7], Jun Wang[1,*]

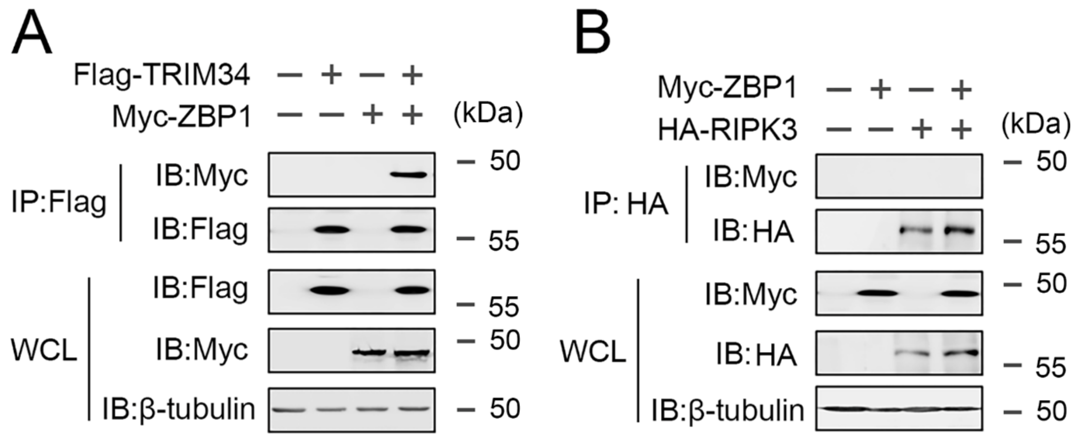


**Figure S1, related to Figure 1. TRIM34 interact with ZBP1.**
(A) 293T cells were transfected with vector, Myc-ZBP1 and Flag-TRIM34. Forty-eight hours post-transfection, Co-IP and immunoblot analysis were performed with the indicated antibodies. (B) Experiments were performed similar to those in (A), except indicated HA-RIPK3 were used. All experiments were repeated at least three times.

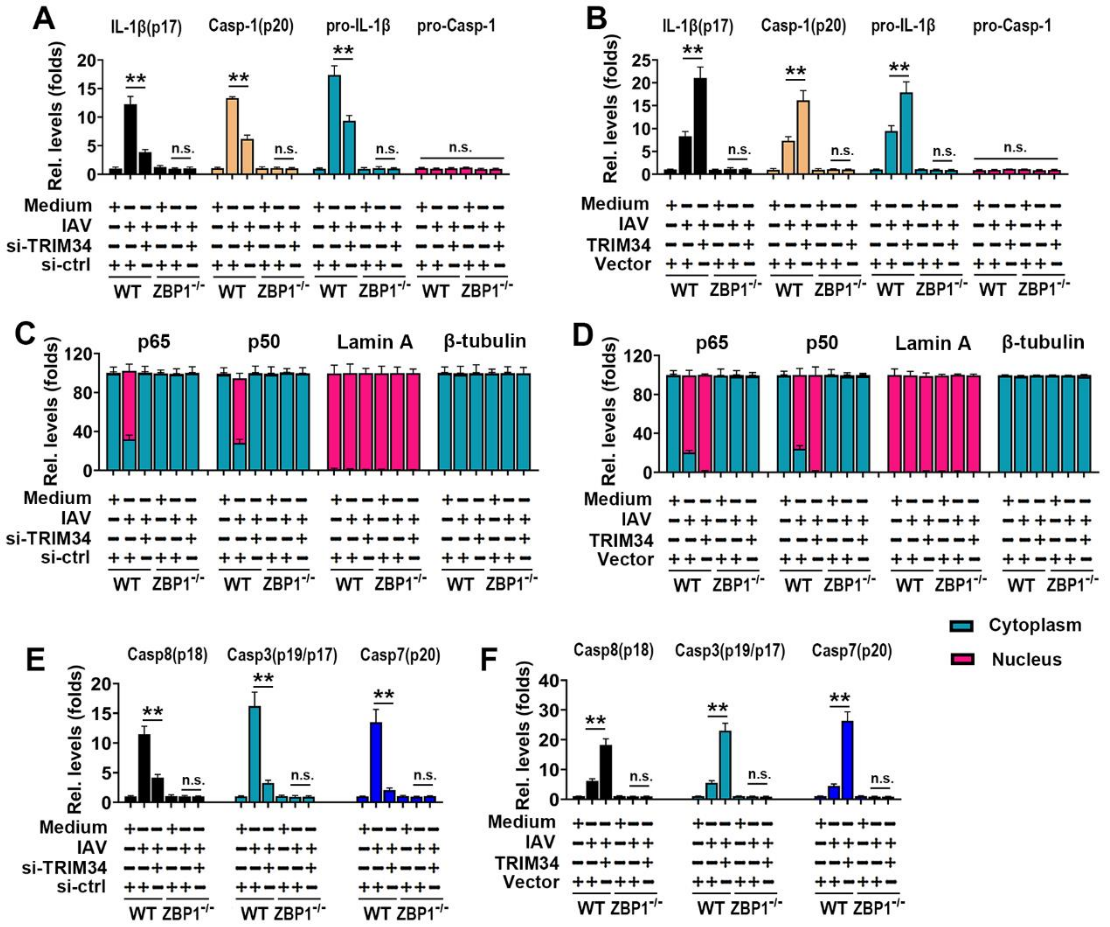


**Figure S2, related to Figure 5. TRIM34 regulates IAV induced NLRP3 inflammasome and programmed cell death pathways via ZBP1 *in vitro*.**

(A and B) The IL-1β (p17)/β-tubulin, Casp-1 (p20)/β-tubulin, pro-IL-1β/β-tubulin and pro-Casp-1/β-tubulin ratios were quantified as described in Fig 5B and 5D.

(C and D) Quantification of p65, p50, Lamin A and β-tubulin cytoplasmic and nuclear localization as described in Fig 5F and 5H.

(E and F) The Casp8 (p18)/Casp8, Casp3 (p19/p17)/Casp3 and Casp7 (p20)/Casp7 ratios were quantified as described in Fig 5J and 5K.

All experiments were repeated at least three times. Bar graphs present means ± SD, n = 3 (**P < 0.01; n.s., not significant).

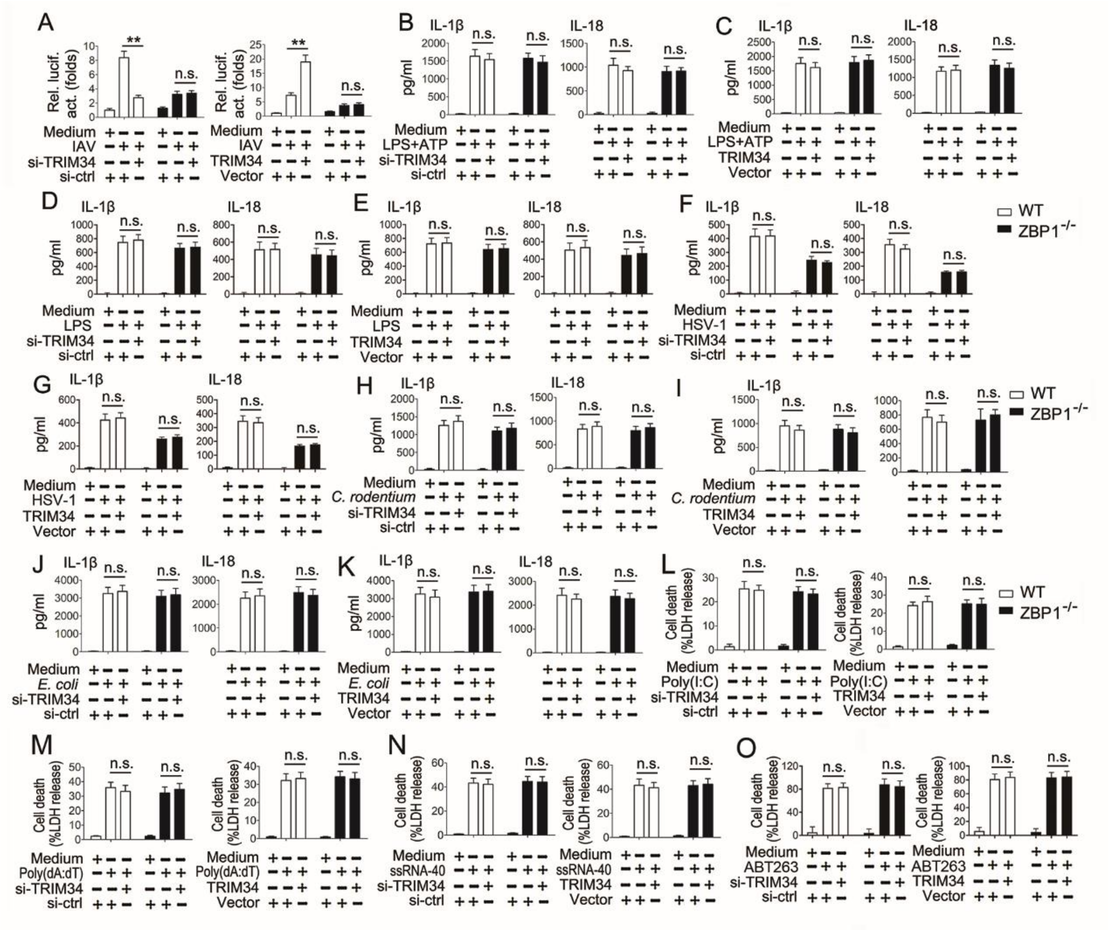


**Figure S3. TRIM34 is dispensable for proinflammatory cytokine production and cell death in response to activation of canonical and non-canonical inflammasomes and transfected RNA ligands.**

(A) THP-1 macrophages were transfected with indicated plasmids, si-RNAs or NF-κB dual luciferase reporter plasmid for 48 h prior to luciferase assays.

(B) THP-1 macrophages were transfected with indicated si-RNAs for 36 h. Then, cells were treated LPS (1 μg/ml for 3 h) and ATP (2.5 mM for 30 min). IL-1β and IL-18 protein levels in cell culture supernatants were determined by ELISA.

(C) Experiments were performed as described in (A), except that cells were transfected with vector control or Flag-TRIM34.

(D-K) Experiments were performed as described in (A), except that cells were treated LPS (1 μg/ml for 6 h) (D and E), infected with HSV-1 (1 MOI for 12 h) (F and G), *C. rodentium* (MOI = 20) (H and I) or *E. coli* (MOI = 20) (J and K) for 20 h.

(L) THP-1 macrophages were transfected with indicated plasmids or si-RNAs for 36 h, and transfected with poly(I:C) (1 μg/ml) for 6 h. Cell death was quantified using LDH release.

(M-O) Experiments were performed as described in (L), except that cell were transfected with poly(dA:dT) (1 μg/ml) for 6 h (M), ssRNA-40 (5 μg/ml) for 24 h (N), or ABT-263 (1 μM) for 72 h (O).

All experiments were repeated at least three times. Bar graphs present means ± SD, n = 3 (**P < 0.01; n.s., not significant).

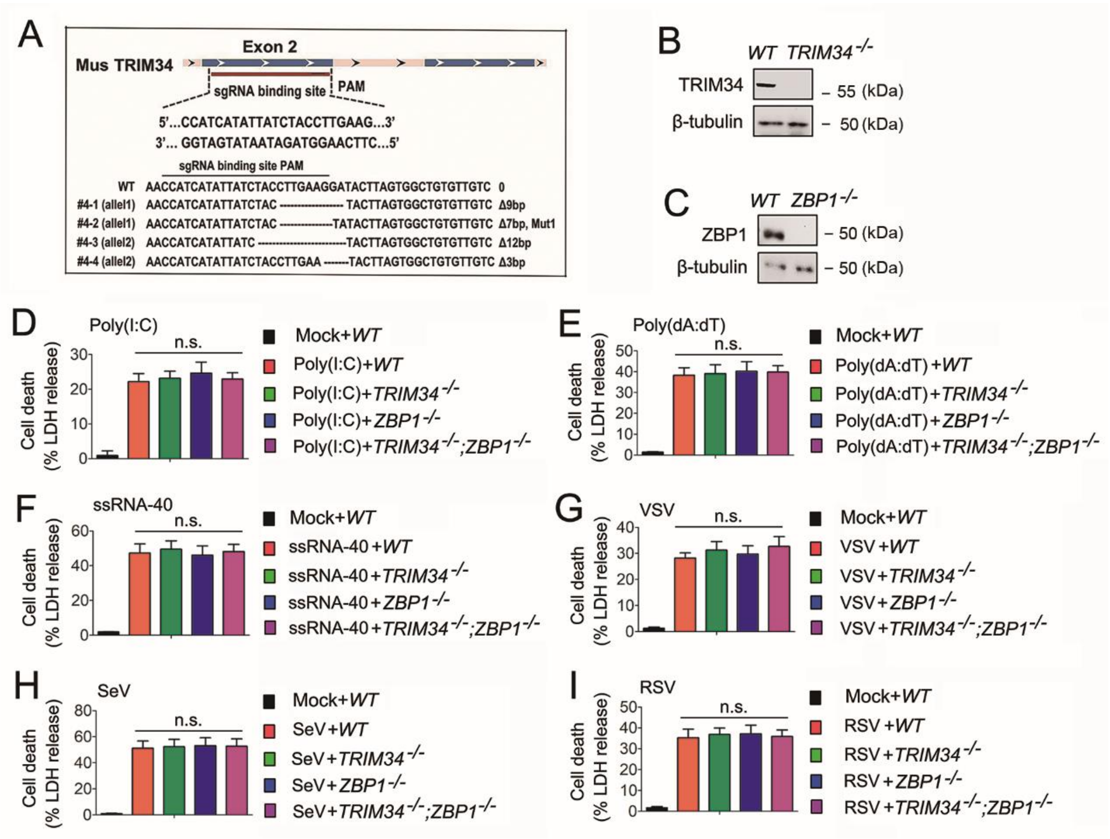


**Figure S4. TRIM34 is dispensable for cell death in response to transfected RNA ligands and RNA viruses belonging to Paramyxoviridae and Rhabdoviridae families.**

(A) schematic showing the single guide RNA (sgRNA)-binding site in the mouse *Trim34* gene (top). Sequence of the genomic single guide RNA-binding site (bottom).

(B) *Trim34* expression in the lung of WT and *Trim34*$^{-/-}$ mice was detected using Western blot analyses.

(C) *ZBP1* expression in the lung of WT and *ZBP1*$^{-/-}$ mice was detected using Western blot analyses.

(D) BMDMs were isolated from indicated mice and transfected with poly(I:C) (1 μg/ml) for 6 h. Cell death was quantified using LDH release.

(E and F) Experiments were performed as described in (D), except that cell were transfected with poly(dA:dT) (1 μg/ml) for 6 h (E), or ssRNA-40 (5 μg/ml) for 24 h (F).

(G) BMDMs were isolated from indicated mice and infected with VSV (MOI=5) for 24 h. Cell death was quantified using LDH release.

(H and I) Experiments were performed as described in (G), except that cell were infected with SeV (MOI = 5) (H), and RSV (MOI = 10) (I) for 24 h.

All experiments were repeated at least three times with consistent results. Bar graphs represent the means ± SEM, n = 3 (**P < 0.01; *P < 0.05).

# Table S1 Antibodies used in this study

| Antibodies | Source | Dilution |
|---|---|---|
| TRIM34 | Abcam (ab180130) | 1:1000 |
| ZBP1 | Abcam (ab180130) | 1:2000 |
| HA | Sigma (H6908) | 1:2000 |
| Flag | Sigma (M2) | 1:2000 |
| Myc | Abcam (ab32) | 1:2000 |
| Caspase 3 | Abcam (ab13847) | 1:5000 |
| Caspase 7 | Abcam (ab 69540) | 1:5000 |
| Caspase 8 | Abcam (ab32397) | 1:5000 |
| Caspase 9 | Abcam (ab202068) | 1:5000 |
| Ubiquitin | Abcam (ab7780) | 1:5000 |
| Ubiquitin(k48) | Abcam (ab140601) | 1:5000 |
| Ubiquitin(k63) | Abcam (ab179434) | 1:1000 |
| Caspase-1 | Abcam (ab138483) | 1:5000 |
| IL-1β | Abcam (ab200478) | 1:500 |
| p65 | Cell Signaling (8242) | 1:2000 |
| p50 | Cell Signaling (3035) | 1:2000 |
| β-actin | Abcam (ab179467) | 1:5000 |
| β-tubulin | Abcam (ab210797) | 1:5000 |
| Lamin A | Abcam (ab26300) | 1:2000 |

# Table S2 Primers used in qRT-PCR

| **Gene name** | **5'primer** | **3'primer** |
|---|---|---|
| **ZBP1** | 5'- CAGGAGTAGATGACAGGGAGTTGC -3' | 5'- CAGGTAGCCATGATGGAAGGTAA -3' |
| **IL-6** | 5'- ACTCACCTCTTCAGAACGAATTG -3' | 5'-AGCCATCTTTGGAAGGTTCAG-3' |
| **IL-1β** | 5'- TGGCAATGAGGATGACTTGTTC -3' | 5'- GCTGTAGTGGTGGTCGGAGATT -3' |
| **TNF-α** | 5'- CCCTGGTATGAGCCCATCTATC -3' | 5'- CTTAGTGGTTGCCAGCACTTCA -3' |
| **β-actin** | 5'- GGACTTCGAGCAAGAGATGG-3' | 5'-AGGAAGGAAGGCTGGAAGAG-3' |
